# The effectiveness of military hospital-based drug treatment program (PMK) when compared with the traditional community-based drug treatment program (FAST)

**DOI:** 10.1186/s12913-019-4479-1

**Published:** 2019-09-10

**Authors:** Niramon Kaewkham, Thawatchai Leelahanaj, Jatsada Yingwiwattanapong, Wanida Rattanasumawong

**Affiliations:** Department of Psychiatry and Neurology, Phramongkutklao Hospital, Royal Thai Army, Bangkok, Thailand

**Keywords:** Addiction, Military, Community-based therapy, Addiction treatment in Thailand

## Abstract

**Background:**

In Thailand, two community-based drug treatment approaches are common. The first one is the traditional community-based treatment program (FAST) which brings the principles of community therapy as a guideline for treatment. The second one is the military hospital-based drug treatment program (PMK), derived from the basic military training, the Buddhist Twelve Steps, CBT and the Minnesota Rehabilitation Model. This study aimed to investigate and compare the efficacy of PMK vs. FAST.

**Method:**

The experiment was conducted from January–March 2016 at the rehabilitation center for patients with drug addiction in Thailand. Quasi-experimental methods were introduced, and ASSIST, WHOQOL-BRIEF-THAI and self-efficacy interview form were applied. After completing the drug rehabilitation program at a total duration of 120 days, the researcher continued at follow up times at 3 and 6 months.

**Results:**

Compared with baseline scores, both programs significantly reduced the severity of drugs and increased self-efficacy at 6-month follow-up. PMK had better improved scores in the relationship and environment dimensions of quality of life at 3-month follow-up (*P* = 0.026, 0.006). The mean quality of life scores in PMK at 3 and 6 months showed better results than mean scores at baseline (*P* = < 0.001).

**Conclusion:**

Both community-based programs in Thailand significantly reduced the severity of drugs and increased self-efficacy scores at 6-month follow-up. PMK and FAST has not shown any significant difference in treatment outcome results in the aspects of self-efficacy and reduced severity of drugs used. However, PMK had significant positive effects on the quality of life.

## Background

Today, drugs have become a major global crisis. In Thailand, the problems have expanded rapidly and have a critical impact on the nation’s economic, social, political, international relations and demographic well-being. Related reports about drug epidemics in the population (ages ranging from 12 to 65 years-old) consisted of ten surveys drug types, including: kratom, marijuana, opium, ecstasy, cocaine, heroin, volatile substances, amphetamines etc... According to data from a household survey in 2011 (the latest study) about 3,500,000 people have used some type of drug, and about 590,000 people have consumed one type of drug yearly [1].

The Thai government has established the Drug Rehabilitation Act B.E. 2545 (2002). This law was proposed to promote measures to help addicts become patients under a legal context [1, 2]. The Act also included consolidating resources related to drug addiction rehabilitation for greater efficiency. However, because drug treatments are costly and inaccessible to the poor, the government suggested the principle provision of community-based rehabilitation support. The main agency responsible is the Ministry of Justice and a key component of this response has been the opening of compulsory centers for treatment and rehabilitation of addicts.

Community-based treatment services are designed to help patients with drug addiction develop the skills to manage their addiction and related problems in the community. Most community-based treatment programs incorporate the following key fundamentals: minimal disruption support systems, comprehensive holistic care and evidence-based practices that are culturally appropriate. In Thailand, two community-based treatment approaches are common. The first one is the traditional community-based treatment program: FAST format Model [3] derived from the Thanyarak Institute bringing the principles of community therapy as treatment guidelines. The second is the military hospital-based drug treatment program of (PMK-Community-Based), derived from the Phramongkutklao Model [4, 5]. Both programs involve drug screening and rehabilitation. Military units of the Royal Thai Army are the main operators. The center provides military officers serving as trainers.

Although most studies have reviewed that community-based therapies helped to reduce the number of people who persistently abused drugs [7, 8] the evidence is still inconsistent. These two approaches are the most commonly available in Thailand, but few studies have investigated their effectiveness regarding substance abusers. Gaining a better understanding of their effectiveness would be important. The first aim of this study was to investigate the effectiveness of both community-based drug treatment programs and the second aim examined the effectiveness of military hospital-based versus original- community-based drug treatment programs.

## Methods

### Participants

All patients receiving rehabilitation services from the three rehabilitation treatment centers in Thailand were enrolled in the study. The experiment was conducted from January to March 2016. Patients were receiving treatment under court order of the Drug Rehabilitation Act B.E. 2545. The patients with drug addiction in this project were aged between 18 and 60. PMK was conducted at the center for rehabilitation of patients with drug addiction, Lat Lum Kaeo District, Pathum Thani, whereas FAST was conducted at Thanyarak Institute’s centers for rehabilitation of patients with drug addiction. The study sample consisted of 176 individuals. In all, 111 patients were treated with the PMK and 65 patients were treated with the FAST programs. In this study, patients with severe psychiatric symptoms, such as acute psychosis or those with mental illness, were excluded from the study. The study was approved by the Ethics Committees of the Royal Thai Army Medical Department. Each subject signed an informed consent form before their research participation.

### Interventions

The FAST Program involves drug rehabilitation treatment over a total duration of 120 days. The content of the drug rehabilitation program comprises 6 topics: 1) basic military support training, 2) training in ideology and democracy, 3) training in health education, 4) training in religion and morals, 5) basic vocational training and 6) FAST Model training [3].

The FAST Model consists of first, family features: (1.1) in this session, the trainer provided family education by creating family activities. This process was implemented by setting up a knowledge base wherein the family members needed to know the attitudes and understanding of the family members’ roles in drug prevention, and skills related to preventing and treating drug abuse at the family level. Then (1.2) family counseling, involved a process bringing the family members together to co-express their feelings, exchange ideas and talk about their experiences together. They also provided support and encouragement to each other, so that members or drug addicts could return to society appropriately. Next, (1.3) the family therapy feature comprised a therapeutic approach combining individual and family therapy in an effort to resolve family conflicts. Second, appropriate alternative treatment methods were applied to rehabilitate the patient, according to the actual conditions of each patient. This activity was intended to provide patients the opportunity to suitably express themselves in a useful way. They also developed their own potential to cultivate love, commitment and responsibility. Finally, this activity could benefit the school, community, society and nation. Third, the self-help process was implemented by providing the patient with training and physical exercise in conjunction with mental and social guidance. This enabled the patient to have strong healing power, as well as good behaviors, attitudes and feelings, resulting in the ability to create a healthy and drug-free environment. Finally, the therapeutic community constituted one of the major components in the rehabilitation process for drug addicts. This process was based on the principle that members could practice self-development. This process was conducted by allowing them to join together as a large family allowing members to develop themselves through a process of changing their negative thoughts to new ones. They could also reflect on the overall physical and mental state of other members of the group [3].

*PMK* comprises a drug treatment program in the Division of Psychiatry and Neurology, Phramongkutklao Hospital constituting a qualified academic unit providing expertise in drug treatment, accepted throughout the country. The rehabilitation camps have been developed using the PMK program, developed from the PMK Model of inpatient drug treatment [4]. In the evolved camp, trainers without basic medical knowledge can use the teachings from this course to rehabilitate and counsel drug addicts, after attending the training center for a period of four months. This course continues to maintain the principles of community therapy, comprising six topics: First, basic military support training, second, training in ideology and democracy, third, training in health education, fourth, training in religion and morals, fifth, basic vocational training and last, the PMK Model.

The PMK Program uses the PMK Model in combination with community-based therapy, while the FAST Program uses the FAST Model together with community-based therapy. The PMK Model is a program of rehabilitation of drug addicts that has been implemented at Phramongkutklao Hospital since 2003. This constitutes an intensive treatment program for patients with drug addiction. In addition, this program emphasizes the Buddhist Twelve Steps, cognitive-behavioral therapy (CBT) and the Minnesota Rehabilitation Model. The main content of the PMK Model was divided into 1) health & psychological education, 2) the Buddhist Twelve steps, 3) relaxation and recreational therapy, 4) CBT and 5) family counseling. The main mechanisms of the PMK Model focus on the motivation to change, drug cessation, spiritual rehabilitation and the Buddhist Twelve Steps. It effectively helps patients to be self-reliant, live a conscious life, knowing and recognizing emotions and the distortion of ideas.

### Measures

The instrument used in this study was “the Alcohol, Smoking and Substance Involvement Screening Test (ASSIST)” [8] used in substance abuse screening and severity of addiction screening; risk levels can be divided in low, moderate and high risk levels. This tool is also used to group the risk of substance abuse occurring during follow-up and after treatment. In addition, this study used the WHOQOL-BRIEF-THAI research tools, [9, 10] used to estimate the quality of life using the World Health Organization standard. The content of the evaluation form is divided in four areas: 1) physical health, 2) psychological health, 3) relationships and 4) the environment. The final tool used in this study was the self-efficacy evaluation questionnaire form [11].

### Data analysis

We implemented a quasi-experimental research design to assess the efficacy of both programs and to comparative efficacy of PMK versus FAST. Participants were monitored at three and six months after joining the rehabilitation program. The researcher followed up with participants by phone, interviewing them from three surveys: ASSIST surveys, QUALITY OF LIFE WHOQOL-BRIEF-THAI surveys and self-efficacy surveys. Data were analyzed using a licensed form of the SPSS Software, Version 21. Descriptive statistics were used for baseline characteristics and demographic data. Between group differences, student *t-*test, chi-square test and the Mann-Whitney U test were used to compare the continuous and categorical variables. The level of statistical significance was kept at *P* < .05.

## Results

### Demographic profile

As a result, the patients in this study totaled 176. In all, 111 patients were treated with PMK, and 65 patients were treated with FAST. All patients were male, aged 26 to 45 years old. Most were single and below bachelor’s’ degree level. The working status of the sample found that most were employed (64%) and earned income lower than THB 15,000 (approximately 78%). We found no significant differences between patients concerning sex, age, income, occupation and chronic illness history education. However, a significant difference was found regarding marital status and education as shown in Table 1.

**Table 1:**
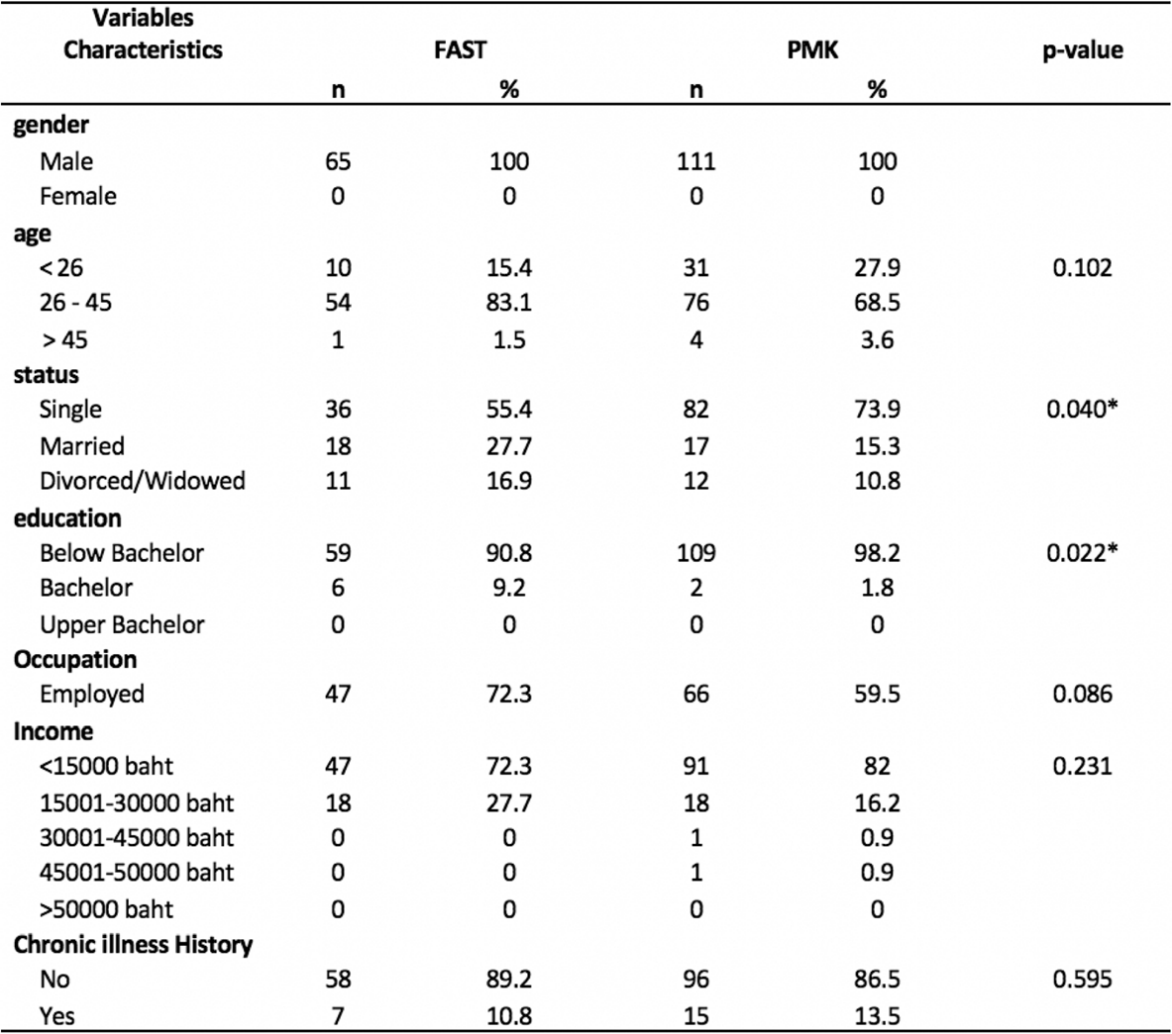
Baseline Demographic and Characteristics of PMK and FAST

*The mean difference is significant at the .05 level

The results showed that the baseline quality of life scores in the group treated with PMK was 90.48%, with a mean ASSIST score of 24.41%. On the other hand, the patients treated with FAST had a baseline quality of life score of 93.12% and a mean ASSIST score of 17.71%. The mean self-efficacy score of patients treated with PMK was 26.95, whereas mean score of patients treated with FAST equaled 27.06. We found no significant differences between groups concerning the baseline quality of life scores and the average score of self-efficacies. However, PMK had a significantly higher average ASSIST score as shown in Table 2.

**Table 2:**
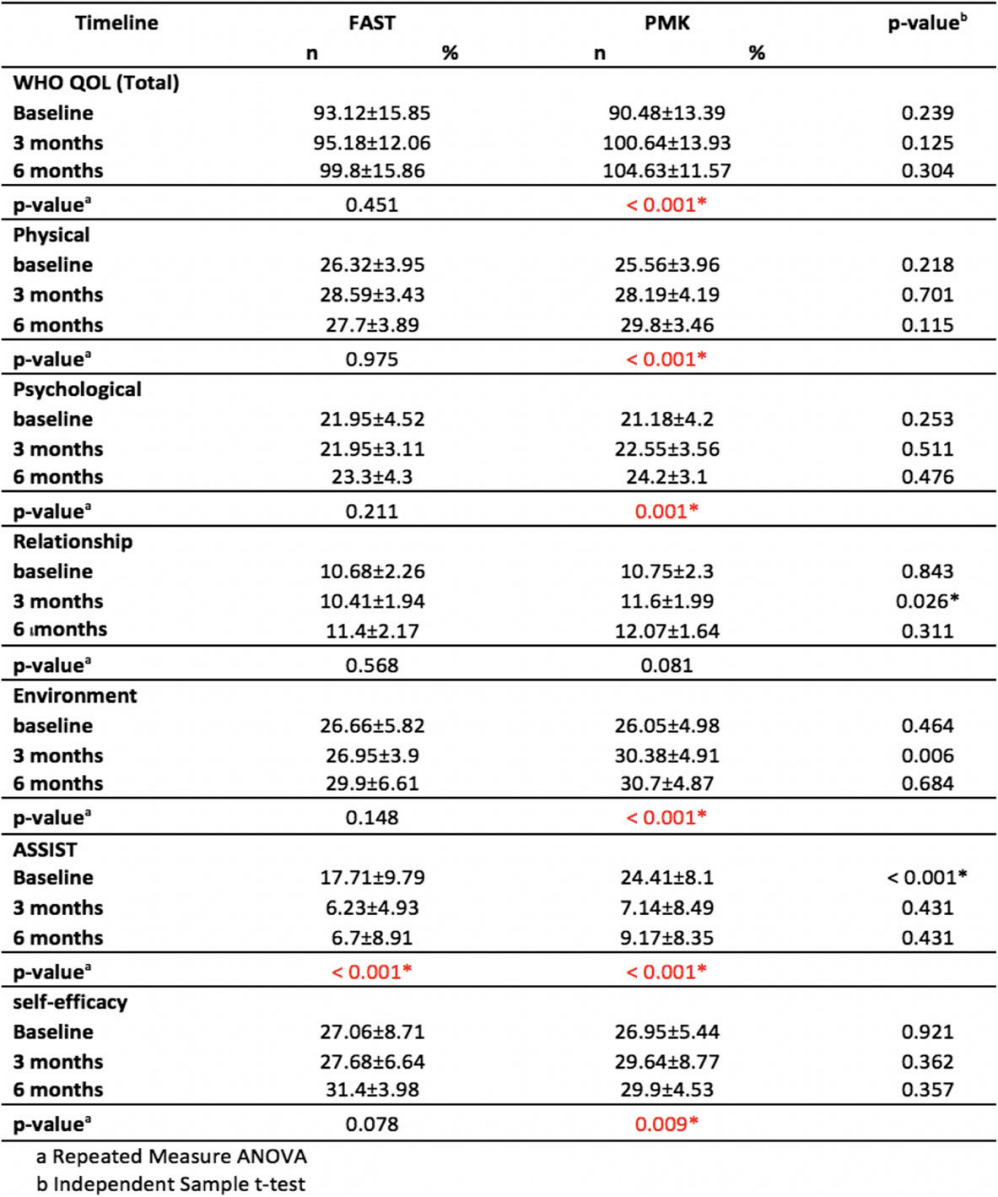
Comparing the mean score of quality of life in several aspects, ASSIST and self –efficacy among PMK versus FAST at baseline, three and six-months follow-up periods

WHOQOL (Total): Quality of life total score

Physical: Quality of life in physical aspect

Psychological: Quality of life in psychological aspect

Relationship: Quality of life in relationship aspect

Environmental Quality of life in environmental aspect

*The mean difference is significant at the .05 level

After completing the three-month treatment program, the researchers were able to follow up with 113 patients, 72 patients treated with the PMK and 41 patients treated with FAST. After completing the six-month treatment program, the researchers were able to follow up with 94 patients, 62 patients treated with PMK and 32 patients treated with FAST.

**Fig. 1:**
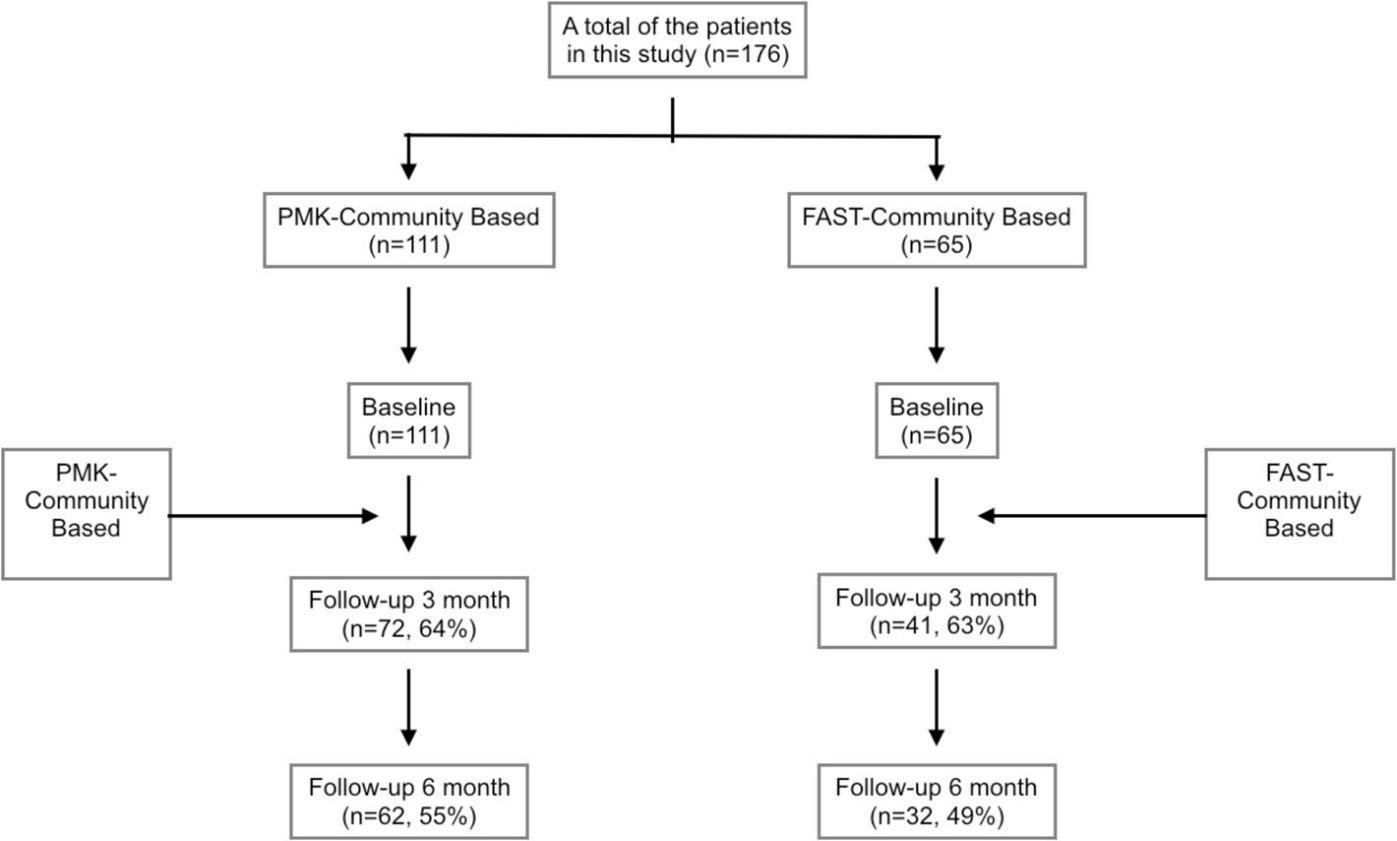
CONSORT flow diagram

Patients receiving PMK remained in treatment longer than youth receiving FAST as shown in Fig. 1.

### Quality of life score

The study results showed an average quality of life mean score, in the group treated with PMK, significantly increased from the mean score at baseline (*P* < 0.001) when compared at three and six months. On the other hand, patients treated with FAST showed no significant increase from baseline. However, when comparing scores between the two groups, no significant difference was observed (Table 2, Fig. 1).

**Fig. 2:**
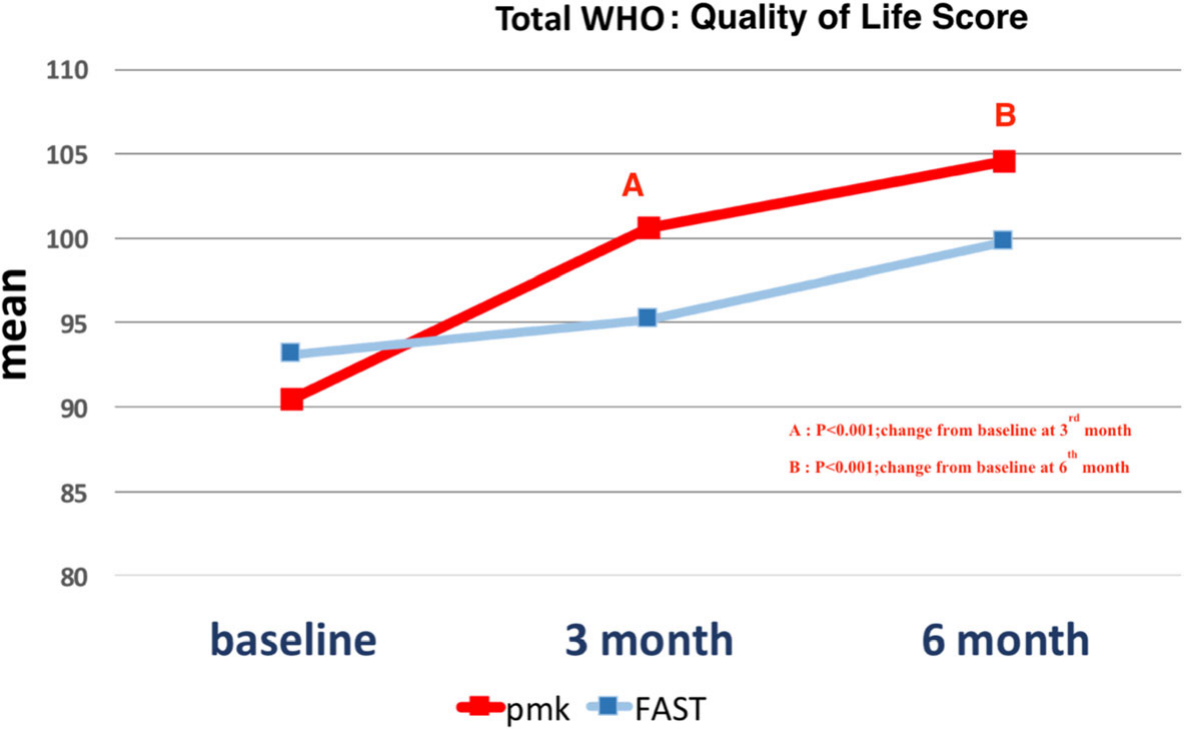
Comparing the quality of life’s mean score between group at three- and six-months follow-up period, the results show the PMK group changed the score significantly more than FAST. At the follow-up time of three months (*P* = 0.02) and six months (*P* = 0.03)

When interpreting scores on quality of life, which were separated in four components, the results of the three-month follow-up period showed that the quality of life in the relationship and environmental aspects of the groups treated with PMK had a mean score significantly greater than FAST (*p* = 0.026, *p* = 0.006. However, no significant difference was found at six months. Regarding the aspects of psychological and physical health, the mean score among groups did not significantly differ at the three- and six-month follow-ups (Table 2, Fig. 2).

### Severity of drugs used

**Fig. 3:**
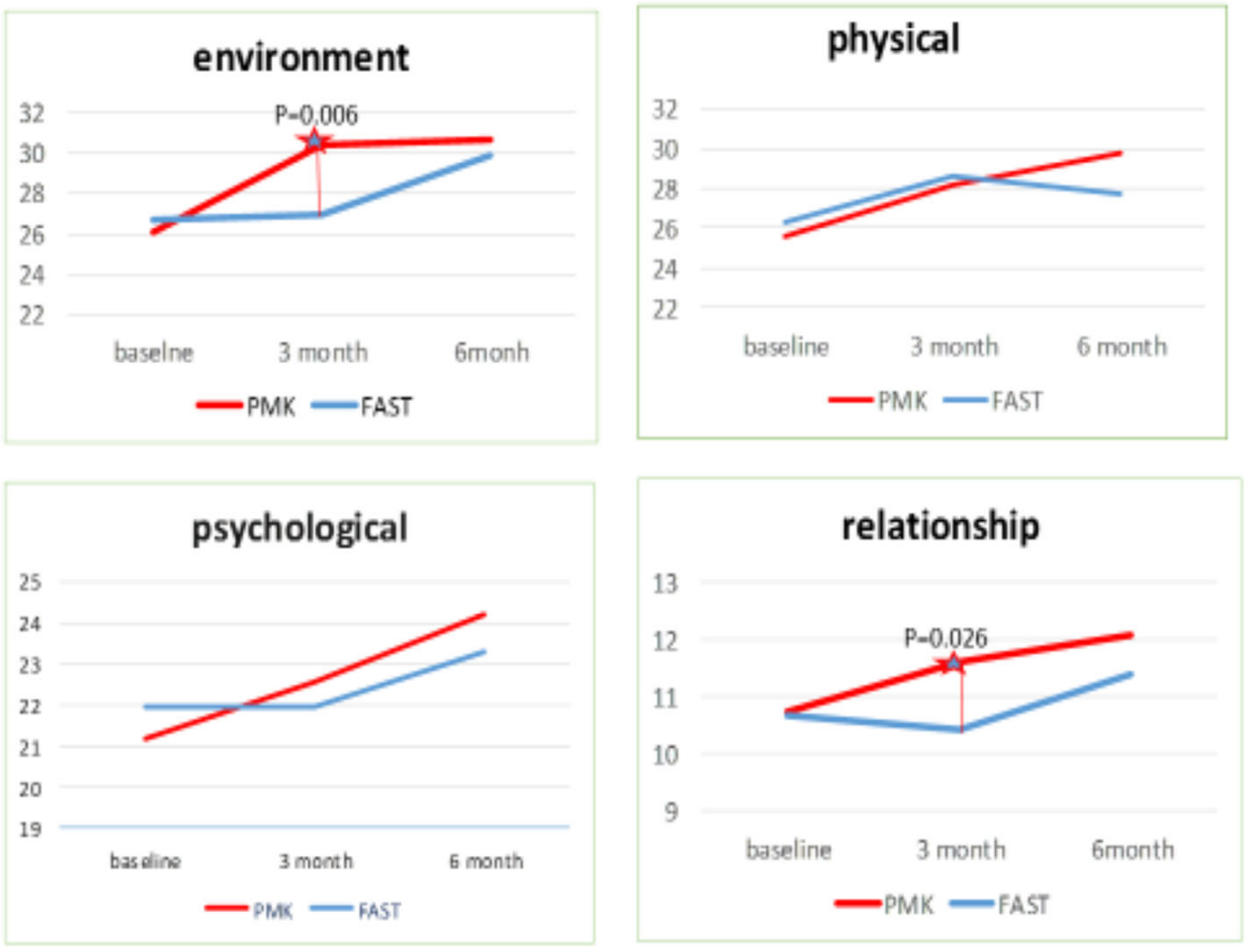
The results of the three-month follow-up period showed that relationship and environmental aspects of the quality of life score in PMK had a mean score significantly greater than FAST (*p* = 0.026, *p* = 0.006). But there was no significant difference in the follow-up period of six months. However, quality of life scores, in the aspects of psychological and physical health, showed the mean score among the groups was not significantly different at the follow-up period of three and six months

The mean score of ASSIST in both groups were significantly lower than the mean score at baseline at both three- and six-month follow-ups (*P* < 0.001) (Table 2, Fig. 3). However, no significant difference was found between the two groups (Table 2).

### Self-efficacy evaluation

The baseline-mean scores of self-efficacy in both group were significantly higher in the two follow-ups of six months (*P* < 0.001, *P* = 0.05) (Table 2).

## Discussion

This paper offers the first assessment of the effectiveness of two well-known community-based programs in Thailand and a secondary assessment of the effectiveness of PMK versus FAST. The evaluation indicated clearly that both programs significantly reduced the severity of drugs and increased self-efficacy scores at six-month follow-up. PMK was effective in improving patient’s quality of life, reducing the severity of drugs used and increasing self-efficacy. PMK also revealed a number of advantages over FAST. The effectiveness of PMK could be proven by the reasons stated below.

PMK uses several processes more than FAST. The first process is CBT. CBT is a powerful process in the treatment of drug users, with extensive evidence-based support for efficacy and benefit [12, 13]. This process helps the patient understand and perceive problems in a realistic way. CBT involves short term psychological rehabilitation emphasizing the treatment psychologically and behavioral problems resulting from negative thinking or abnormal thinking processes. Therefore, patients’ thoughts, beliefs and perceptions are made congruent to reality and positive to themselves. In this context, the repetitive use of drugs does not mean failure. Similarly, the study of Zhuang et al. [12] indicated that cognitive behavioral intervention has the effect of correcting the mind, reducing negative symptoms, creating a better lifestyle and improving quality of life. Moreover, the research also found that cognitive behavioral intervention improves the quality of life, specifically, of patients with heroin addiction.

The second process involves the Twelve Steps concept of Buddhism [5] adapted from the Western principle of the twelve-step concept. Thai culture is strongly rooted in spiritual and religious traditions. The principles of the Buddhist Twelve Steps emphasize faith, spirituality, mindfulness and wisdom, as well as a higher power, i.e., the Buddha, Dharma, and Sangha and these functions as a protective factor. This method helps to create a new way of life that is mindful and spiritual. Moreover, the Buddhist Twelve Steps rely on these principles of self-value and a higher power to solve problems they are encountering as well as to have a real peaceful and happy life and be able to support themselves and their family. It has been used as an important guide to rehabilitate patients in Phramongkutklao Hospital [5]. In addition, this approach is consistent with Thai characteristics and Thai culture, helping to reach patients across all states of physical, mental, and spiritual health, as well as social/societal strata [5].

Finally, PMK relies on group processes and motivational enhancement. Patients treated with the PMK were involved in group treatment activities using motivational enhancement skills, while the traditional treatment program did not focus on group processes. The group-based treatment developed interpersonal relationship skills on the basis of mutual trust and faith that helped patients to develop skills to refuse in a reasonable manner. Addicts experience the stigma of being a drug addict, as well as lack confidence and self-esteem, and express feelings of separation and guilt. Group treatment has a number of prominent factors that can alleviate such feelings. These consist of helping the patient feel less stigmatized, support his peers and exemplify to other members who are learning new problem-solving skills, exchanging information and helping each other. The patients can confront problematic behaviors, clarify problems and expose their feelings. Another point to consider is how this strategy can be used to give members a good experience in therapy sessions.

For example, Pothirat et al. [14] studied the effects of behavioral group therapy in comparison with an education program on smoking cessation behavior. The result showed that behavioral group therapy was more successful than the education program. In addition, Flores et al. asserted in his research that “group therapy” is a treatment of choice for addiction [15].

The ASSIST and self-efficacy scores between the two groups did not significantly differ. When we focused on the results for each group, we found that patients in both treatment programs showed improvement from baseline. This may explain by both programs used community therapy in their processes, producing nearly the same effect. The community therapy process enables members to develop the life skills necessary to empower themselves and move towards a drug-free life. The results conform to the literature research review of Malivert et al. [6], concerning the topic of community effectiveness. Malivert found this method reduced the re-use of drug substances. In addition, citing the study of Sadir et al. [16], They found that community therapy has many beneficial effects for the patients in the areas of mental health, physical health and social function.

## Limitations

Firstly, no randomize sampling was conducted of the study population, which may have affected the data and led to biased results. Secondly, the subjects in the present study did not include female patients as a result, the generalizability of the findings seemed to indicate a limited group population. Thirdly, patients with drug addiction had a high “drop-out” rate. This made it impossible to track data completely. Therefore, if the research data collection system could be improved, in collaboration with the original agencies responsible for patient care at the outset point, it should be possible to obtain more, and create more, accurate and credible information. Increasing the number of participants would also make the data more accurate. Lastly, the manual used in the study was developed and applied for the first time. Thus, further evaluation and improvement should be conducted to ensure its effectiveness.

## Conclusion

Both community-based programs in Thailand significantly reduced the severity of drugs and increased self-efficacy scores at six-month follow-up. PMK and FAST did not indicate any significant difference in treatment outcomes or results in the aspects of self-efficacy and reduced severity of drugs used. However, PMK revealed significant positive effects on the quality of life.

## Data Availability

The datasets and materials during the current study are available from the corresponding author on reasonable request.

## Abbreviations

PMK: Military Hospital-Based, Phramongkutklao Model- Based
FAST: Original- Community-Based Drug treatment program
ASSIST: Alcohol, Smoking, and Substance Involvement Screening Test
WHOQOL-BRIEF-THAI: World Health Organization Quality of Life Instruments, Thai version
